# Disrupting the α7nAChR–NR2A protein complex exerts antidepressant-like effects

**DOI:** 10.1186/s13041-021-00817-3

**Published:** 2021-07-05

**Authors:** Anlong Jiang, Ping Su, Shupeng Li, Albert H. C. Wong, Fang Liu

**Affiliations:** 1 Campbell Family Mental Health Research Institute, Centre for Addiction and Mental Health, 250 College Street, Toronto, ON M5T 1R8 Canada; 2 Departments of Pharmacology, University of Toronto, Toronto, ON M5T 1R8 Canada; 3 Institutes of Medical Science, University of Toronto, Toronto, ON M5T 1R8 Canada; 4 University of Toronto, Toronto, ON M5T 1R8 Canada; 5 University of Toronto, Toronto, ON M5T 1R8 Canada

**Keywords:** α7 Nicotinic acetylcholine receptor (α7nAChR), Extracellular signal-regulated kinase (ERK), Forced swim test (FST), Major depressive disorder (MDD), NMDA glutamate receptors (NMDARs)

## Abstract

Major depressive disorder (MDD) is associated with significant morbidity and mortality. Most antidepressant medications target the serotonin and norepinephrine transporters, but a significant minority of patients do not respond to treatment and novel therapeutic targets are needed. We previously identified a protein complex composed of the α7 nicotinic acetylcholine receptor (nAChR) and NMDA glutamate receptors (NMDARs), through which α7nAChR upregulates NMDAR function. Disruption of the α7nAChR–NMDAR complex with an interfering peptide blocked α7nAChR-mediated upregulation of NMDAR function and cue-induced reinstatement of nicotine seeking in rat models of relapse. Here we report that disrupting the α7nAChR–NMDAR complex with the interfering peptide also has antidepressant-like effects in the forced swim test (FST), a common rat behaviour screening test for antidepressant effects. Furthermore, the interfering peptide significantly increases extracellular signal-regulated kinase (ERK) activity in the animals subjected to the FST. Our results provide a novel potential therapeutic target for the development of new antidepressant medications.

**Supplementary Information:**

The online version contains supplementary material available at 10.1186/s13041-021-00817-3.

## Introduction

Major depressive disorder (MDD) is a leading cause of morbidity and mortality, affecting approximately 216 million people worldwide (3% of the global population), which is an increase of 17.8% from 2005 to 2015 [1]. Current pharmacotherapy for MDD includes antidepressant medications in several major categories: selective serotonin reuptake inhibitors (SSRIs) [2, 3], tricyclic antidepressants (TCA) [4, 5], and monoamine oxidase inhibitors (MAOIs) [6–8]. Several types of psychotherapy are also commonly employed to treat MDD: cognitive-behavioral therapy (CBT) [9], interpersonal therapy (IPT) and traditional psychodynamic psychotherapy [10, 11]. Less common but highly effective brain stimulation treatments are also used, and electroconvulsive therapy is the oldest and most well-known [12].

Antidepressant medications are more effective than placebo but overall effects are modest [13]. Although 67% of patients improve with antidepressant treatment, the significant minority of non-responders in a very common disorder results in a great unmet need for treatment in a large number of patients [14]. Other neurotransmitter systems have also been implicated in mood regulation and antidepressant treatment, and a brief review of salient findings follows.

In 1972 the cholinergic hypothesis was proposed by Janowsky and colleagues, who suggested that depressive symptoms are associated with hyperactive cholinergic neurotransmission [15]. Early observers noted that inhibition of acetylcholine esterase (AChE) by organophosphate poisoning led to depressive symptoms and people using these substances often had higher rates of depression. Follow-up experiments also showed that physostigmine, a AChE inhibitor that can penetrate the brain-blood-barrier, increased depressive symptoms and decreased mania [16]. Conversely stressors such as electric shock or forced swimming that can induce depressive-like behaviours also sensitize the cholinergic system [17].

Nicotinic acetylcholine receptor (nAChR) antagonists and partial agonists can have anti-depressive effects [18]. Mecamylamine is a non-selective and non-competitive inhibitor of nAChRs, that has anti-depressant-like effects on the forced swim test (FST) and tail suspension test (TST) [19]. These anti-depressive effects were abolished in mice with β2 or α7 subunit knock-out [19], suggesting that the α7nAChR receptor is necessary for the anti-depressant effects of mecamylamine.

The recent approval of esketamine nasal spray for treatment-resistant major depression by the American Food and Drug Administration highlights the importance of the glutamate system in modulating depressive symptoms [20]. Ketamine is a potent non-selective NMDA allosteric inhibitor that has been used for decades as an anesthetic, often for pediatric and veterinary applications, but the anesthetic dose is much higher than antidepressant doses [21–23]. Compared to conventional antidepressants that require weeks to take effect, ketamine has almost immediate antidepressant effects [24–27]. Ketamine is also a relatively common recreational hallucinogenic street drug, which has hampered research into therapeutic use as an antidepressant. Because of the hallucinogenic effects, abuse potential and associated side effects, ketamine and esketamine are typically administered to treatment-resistant patients under medical supervision [28].

In addition to antidepressant effects of ketamine, there is evidence that the glutamate system is dysregulated in depression. There are elevated levels of glutamate in plasma and cerebrospinal fluid (CSF) from depressed patients compared to unaffected control subjects [29–31], that is normalized by antidepressant treatment [29, 32]. Similar results are seen in post-mortem studies, with elevated glutamate levels in the prefrontal cortex (PFC) of people with MDD and bipolar disorder [33, 34].

The protein expression levels of the glutamate receptor subunit NR2A are increased in the lateral amygdala, and NR2C protein expression is increased in the locus coeruleus [35, 36]. Although suicide is associated with many different mental illnesses, depression is the most common. Suicide victims have high mRNA expression levels of NR2B and NR2C in locus coeruleus neurons [37]. However, in the PFC of depressed subjects, decreased expression of NR2A and NR2B subunits was discovered [38]. There is also an upregulation of NMDA receptor mRNA (GRIN1, GRIN2A–D) and glutamate related genes in the dorsolateral PFC of female MDD patients [39].

Our lab has previously demonstrated that the α7nAChR forms a protein complex with the NR2A subunit of the NMDA receptor that is involved in nicotine addiction. A peptide that blocks the α7nAChR–NR2A interaction also blocks cue-induced reinstatement of nicotine self-administration [40]. Furthermore, activation of α7nAChR increases NMDA-mediated whole cell currents and this is abolished by the α7nAChR–NR2A interfering peptide [41]. Because the glutamate system modulates mood and depression, we hypothesized that the α7nAChR–NR2A interfering peptide might have antidepressant effects. We tested this hypothesis with a common rodent behaviour test for antidepressant-like effects, the forced swim test. In addition to detecting antidepressant-like effects, we found that the α7nAChR–NR2A interfering peptide also increased phosphorylation of extracellular signal-regulated kinase 1 (ERK1). These results suggest that the α7nAChR–NR2A protein complex may be a new therapeutic target for developing novel antidepressant medications.

## Materials and methods

### Animals

Male Sprague–Dawley rats, weighing between 200 and 225 g, were purchased from Charles River Laboratory (Wilmington, MA) and allowed to acclimate to our animal colony for 1 week. The animals were housed in a controlled environment where the temperature was maintained between 20 and 23 °C with a 12-h day-night cycle (7 AM–7 PM). They were housed in pairs initially and then single housed after surgery. The animal experiments in this paper were approved by the Animal Care Committee at Centre for Addiction and Mental Health (Toronto, Canada).

### Drugs and administration

The TAT-peptide was chemically synthesized and purchased from Gen Script (New Jersey, USA) with > 95% purity. The sequence of the peptide is YGRKKRRQRRRR. The interfering peptide, TAT-α7-peptide, was chemically synthesized and purchased from Biomatik (Cambridge, ON) with > 90% purity. The sequence of the peptide is YGRKKRRQRRRRLNWCAWFLRM. Both the TAT-peptide and the TAT-α7-peptide were dissolved in filtered saline to a final concentration of 10 mM. The stock solution was aliquoted and stored at − 80 °C. Imipramine (Sigma-Aldrichs) was dissolved in filtered saline on the day of injection at a concentration of 10 mg/mL.

### Surgical implantation of cannulae

A cannula (HRS Scientific) was surgically implanted into the right lateral ventricle under stereotaxic guidance. On the day of surgery, the rat was anaesthetized with 5% (v/v) isoflurane (Baxter Corporation) in the induction chamber before being placed into the stereotaxic frame. Anaesthesia was maintained with 2% (v/v) isoflurane. Carprofen (Pfizer) (5 mg/kg) was diluted with saline and subcutaneously injected to the incision site for analgesia. The following flat skull coordinates were used to locate the right cerebral ventricle: 1.0 mm posterior to Bregma, 1.4 mm lateral to the midline and 3.6 mm ventral to the surface of the skull. The cannula was slowly lowered into place and secured with three stainless steel screws and dental cement, and a dummy (HRS Scientific) was inserted into the canula to prevent clogging. After surgical procedures, the rats were given 1 mL of saline subcutaneously to prevent dehydration and allowed to recover for a week with wet mash and extra water.

### Interfering peptide delivery

TAT, TAT-α7-peptide, or saline were delivered via* i.c.v.* injection through the implanted cannula. The concentration of each peptide was 1 nmol/μL and different volumes of peptide solution (TAT or TAT-α7-peptide) were injected with two different dose regimens. The first regimen consists of three injections: immediately after FST pre-test, and 5 h and 1 h before the behavioural tests. The second regimen was a single injection 1 h prior to the FST. All peptides were injected with a 10 μL syringe (Hamilton) over 2 min, with a maximum volume of 4 μL. Once the injection was complete, the injector was left in place for 2 min to prevent backflow, then the dummy was replaced. After peptide injection, animals were closely monitored for adverse effects q1hr for 4 h. Imipramine was used as a positive control at a dose of 20 mg/kg, i.p., delivered on the same schedule as the peptide.

### Forced swim test (FST)

The FST has two phases, the pre-test and the swim, with a 24-h time interval between the two. For both phases, the apparatus consisted of an acrylic cylinder 60 cm high and 20 cm in diameter, filled 40 cm deep with water at 23–25 °C. In this configuration, rats are unable to escape from the top of the cylinder, nor are they able to touch the bottom, and thus are forced to swim. In the pre-test, rats are forced to swim for 15 min, after which they were hand-dried and placed under a heat lamp for at least 15 min before receiving the peptide or control injection. 24 h later, rats were forced to swim for 5 min. All tests were carried out in the same room and were video recorded to determine the immobility time.

### Locomotor activity test

Locomotor activity was measured to assess possible confounding effects on the FST. Hyperactivity could obscure antidepressant-like effects due a non-specific reduction in immobility and could also produce a ceiling effect in which high activity cannot increase much further and thus mask changes on the FST. After the FST, the rats were allowed to recover for a week, and all animals were kept in the same treatment groups as FST. Before measuring locomotor activity, the peptides (TAT, TAT-α7-peptide or saline) were injected *i.c.v.* following the same dose regimen as the FST. Each animal was placed in a 20 cm high, 20 cm wide and 30 cm long cage. Locomotor activity was detected by the interruption of horizontal light beams that form a grid just above the cage floor. Activity was recorded for one hour at 5-min intervals.

### Brain tissue collection

Animals were sacrificed one week after the locomotor test. One hour prior to sacrifice, the rats received the same amount of either TAT or TAT-α7-peptide, and saline. The rats were anaesthetized by 5% isoflurane in the chamber and then decapitated with a guillotine. Three different brain regions including the PFC, hippocampus, and striatum were immediately dissected, placed on ice and stored at − 80 °C for further biochemical analysis.

### Co-immunoprecipitation and Western blot

Co-immunoprecipitation and Western blot analyses were performed as previously described [40, 42–44]. All brain tissue samples from rats were homogenized in lysis buffer (50 mM Tris, 150 mM NaCl, 2 mM EDTA, 1% NP-40, 0.5% sodium deoxycholate, pH = 7.4) supplemented with a protease inhibitor cocktail (Sigma-Aldrich). The protein concentration in each sample was quantified by bicinchoninic acid (BCA) assay (Pierce). For co-immunoprecipitation, 500 μg solubilized protein was extracted from whole brain, and incubated with 25 μL protein A/G plus agarose (Santa Cruz Biotechnology) for 1 h at 4 °C, followed by the addition of primary antibody or control IgG (2 μg) with freshly washed 25 μL protein A/G plus agarose overnight at 4 °C. Pellets were washed, boiled for 8 min in SDS sample buffer, and subjected to SDS-PAGE. Total protein extract (50–100 μg) was used as a control in each experiment. After transfer of proteins into nitrocellulose, membranes were subjected to Western blot with the primary antibodies. The protein level was quantified by densitometry (rats: ImageLab, Bio-Rad). The antibodies used were: anti-α7nAChR (1:1000, Santa Cruz Biotechnology, rat), anti-NR2A (1:1000, Novus Biologicals, rabbit), anti-α-Tubulin (1:20,000, Sigma-Aldrich, mouse), anti-p44/42 MAPK (ERK1/2) (1:1000, Cell Signaling Technology, rabbit), anti-P-p44/42 MAPK (T202/Y204) (1:1000, Cell Signaling Technology, rabbit), anti-BDNF (1:1000, Abcam, rabbit), horseradish peroxidase (HRP)-conjugated secondary antibodies against the light chain of rabbit IgG (1:5000, Abcam), and HRP-conjugated secondary antibodies (1:10,000, Cell Signaling Technology).

### Statistical analyses

Densitometric analysis of Western blots was performed by ImageLab (Bio-Rad Laboratory) and videotaped behavioural tests were analyzed manually. Results are presented as the mean ± standard error of the mean (SEM) and dots on the bar display each data point. For group comparisons, Student’s t-test were performed. One-way or two-way ANOVA (Graphpad Prism) followed by the post hoc Tukey test for multiple testing was used for multiple group comparisons in all other experiments.

## Results

### The TAT-α7-peptide has antidepressant-like effects in the forced swim test

**Fig. 1:**
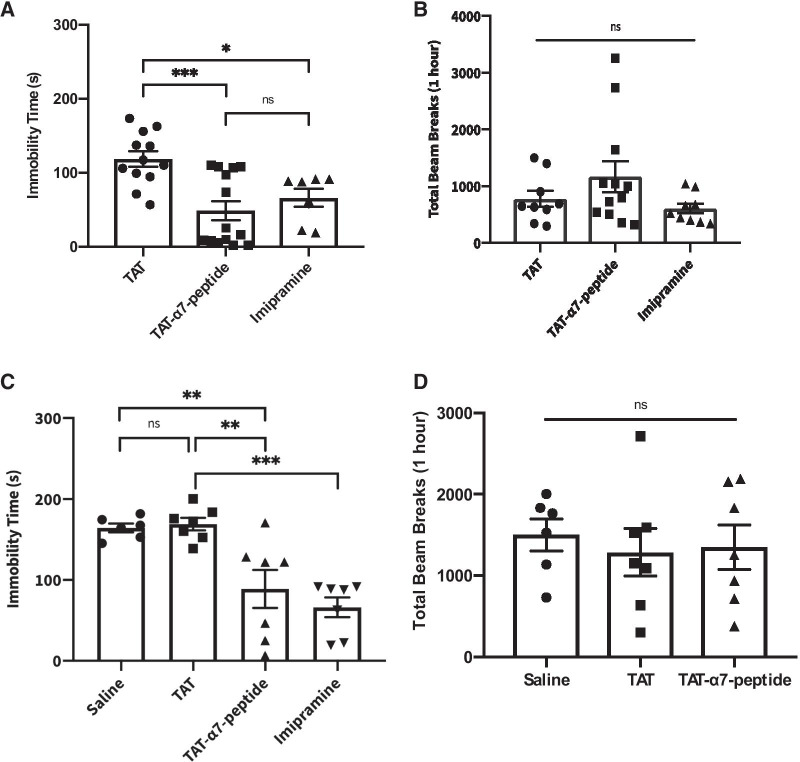
Three i.c.v. injections of TAT-α7-peptide reduces FST immobility time without altering locomotor activity. **A** The immobility time of animals that received three injections (4 nmol of TAT or TAT-α7-peptide i.c.v., and 20 mg/kg imipramine i.p.) in the FST. The drug was injected immediately after pre-test, 5 h before actual swim test and 1 h before actual swim test. FST was performed 1 h after the last injection and the test was video recorded. Immobility time for each animal was recorded manually. Data are presented as mean ± SEM and were analyzed using one-way ANOVA followed by Tukey’s post hoc test. *ns* no statistical significance, *p < 0.05, ***p < 0.001 as compared to TAT group. n = 12 (TAT)/14 (TAT-α7-peptide)/7 (imipramine). **B** Locomotor activity of animals that received three injections (4 nmol of TAT or TAT-α7-peptide i.c.v., and 20 mg/kg imipramine i.p.). The drug was injected 24 h, 5 h and 1 h before the locomotor test. Locomotor activity was measured after 1 h of the last injection. The locomotor activity was measured by total beam breaks in a 1-h time interval. Data are shown as mean ± SEM and were analyzed using one-way ANOVA followed by Tukey’s post hoc test. *ns* no statistical significance. n = 12 (TAT)/14 (TAT-α7-peptide)/7 (imipramine). **C** The immobility time of animals that received a single injection (4 nmol of TAT or TAT-α7-peptide i.c.v., saline i.c.v., and 20 mg/kg imipramine i.p.) in the FST. FST was performed 1 h after the last injection and the test was video recorded. Immobility time for each animal was recorded manually. Data are presented as mean ± SEM and were analyzed using one-way ANOVA followed by Tukey’s post-hoc test. *ns* no statistical significance as compared to saline group, **p < 0.01, ***p < 0.001 as compared to TAT group. n = 6 (Saline)/7 (TAT/TAT-α7-peptide). **D** Locomotor activity of animals that received a single injection (4 nmol of TAT or TAT-α7-peptide ICV, and saline ICV). The drug was injected 1 h before testing locomotor activity, which was measured by total beam breaks in a 1-h time interval. Data are presented as mean ± SEM and were analyzed using one-way ANOVA followed by Tukey’s post hoc test. *ns* no statistical significance. n = 6 (Saline)/7 (TAT/TAT-α7-peptide)

To test potential antidepressant-like effects of the α7nAChR–NR2A interfering peptide, we delivered three doses at 4 nmol. The experimental setup and dose regimens were based on previous studies [40, 43]. Immobility time on the FST was the dependent variable compared between each treatment group: TAT (intracerebroventricular, i.c.v., 4 nmol, n = 12), TAT-α7-peptide (i.c.v., 4 nmol, n = 14) and imipramine (intraperitoneal, i.p., 20 mg/kg, n = 7). As shown in Fig. 1A, imipramine, the positive control antidepressant medication, had the expected effect of significantly lower FST immobility time compared to the TAT control group (one-way ANOVA, F_2,30_ = 9.705, p < 0.05, post hoc Tukey’s HSD, TAT vs. TAT-α7-peptide: p < 0.001; TAT- α7-peptide vs. Imipramine: p > 0.05). The TAT-α7-peptide also significantly reduced immobility time in the FST (p < 0.001), similar to the effect of imipramine.

Because the FST relies on motor behaviour, the results can be confounded by stimulant or sedative effects of medications. Thus, we assessed locomotor behaviour and found that TAT-α7-peptide did not significantly alter locomotor activity. We compared the total beam breaks in a 1-h interval, between TAT-α7-peptide, TAT control and imipramine groups (Fig. 1B, One-way ANOVA, F_2, 27_ = 2.036, p > 0.05, post hoc Tukey’s HSD; TAT vs. TAT-α7-peptide: p > 0.05; TAT-α7-peptide vs. Imipramine: p > 0.05).

### A single dose of TAT-α7-peptide has antidepressant-like effects

**Fig. 2:**
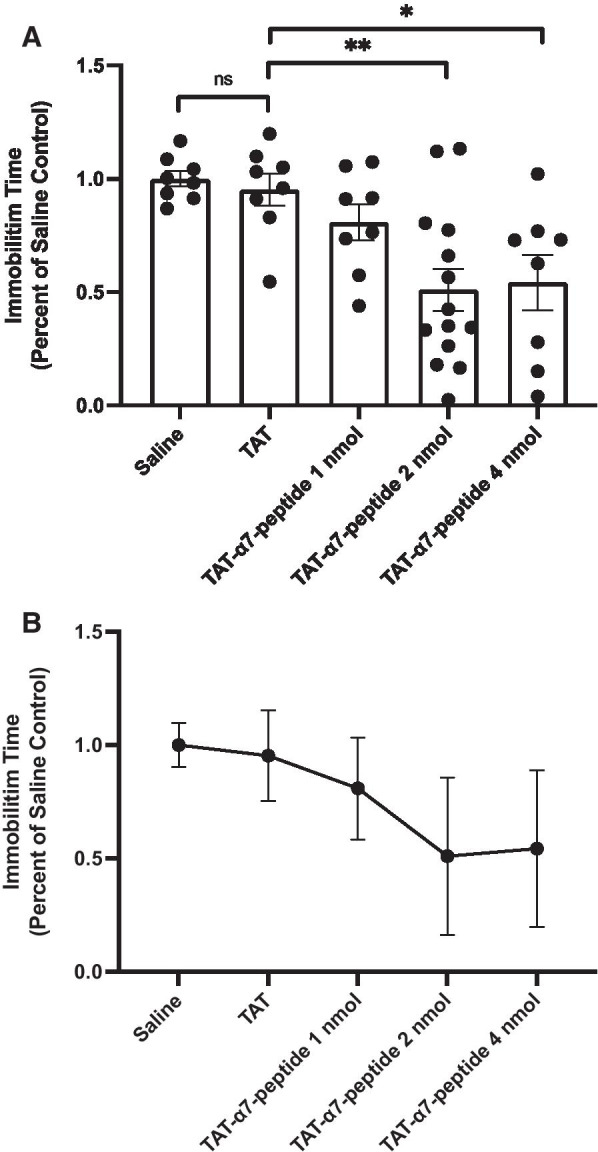
Immobility time of different dosage of interfering peptide. **A** The immobility time of animals that received a single injection (4 nmol, 2 nmol and 1 nmol of TAT or TAT-α7-peptide i.c.v., and saline i.c.v.) in the FST. FST was performed 1 h after the last injection and was video recorded. Immobility time was recorded manually. Data are presented as mean ± SEM and were analyzed using one-way ANOVA followed by Tukey’s post hoc test (*p < 0.05, **p < 0.01). n = 14 for 2 nmol group and n = 8 for the rest of the groups. **B** Dose response curve of the interfering peptide on FST immobility time (single injection: 4 nmol, 2 nmol and 1 nmol of TAT or TAT-α7-peptide i.c.v., and saline i.c.v.). FST was performed 1 h after the last injection and the test was video recorded. Immobility time was recorded manually. Data are presented as mean ± SEM. n = 14 for 2 nmol group and n = 8 for the rest of the groups

Since the three dose regimen had antidepressant-like effects in the FST, we also tested the effect of a single dose of the TAT-α7-peptide. As shown in Fig. 2A, a single dose of TAT-α7-peptide significantly reduced immobility time compared to saline or TAT (Fig. 1C, One-way ANOVA, F_3, 23_ = 13.01, p < 0.0001, post hoc Tukey’s HSD; saline vs. TAT-α7-peptide: p < 0.01; TAT vs. TAT-α7-peptide: p < 0.01; TAT-α7-peptide vs. imipramine: p > 0.05; TAT vs. imipramine: p < 0.001), without causing an increase in locomotion (Fig. 1D, One-way ANOVA, F_2, 17_ = 0.1629, p > 0.05, post hoc Tukey’s HSD; saline vs. TAT-α7-peptide: p > 0.05; TAT vs. TAT-α7-peptide: p > 0.05). These results demonstrate that a single injection of the interfering peptide is sufficient for antidepressant-like effects in the FST.

We further tested the antidepressant-like effect of the interfering peptide at various doses (1, 2, and 4 nmol). As shown in Fig. 2A, FST immobility time was decreased significantly by both 2 nmol and 4 nmol of the TAT-α7-peptide (One-way ANOVA, F_4, 41_ = 6.591, p < 0.01, post hoc Tukey’s HSD; saline vs. 4 nmol TAT-α7-peptide: p < 0.05; TAT vs. 4 nmol TAT-α7-peptide: p < 0.05; saline vs. 2 nmol TAT-α7-peptide: p < 0.01; TAT vs. 2 nmol TAT-α7-peptide: p < 0.01), but not 1 nmol TAT-α7-peptide. A dose response curve is shown in Fig. 2B.

### Disruption of α7nAChR–NR2A interaction by TAT-α7-peptide in rat hippocampus

**Fig. 3:**
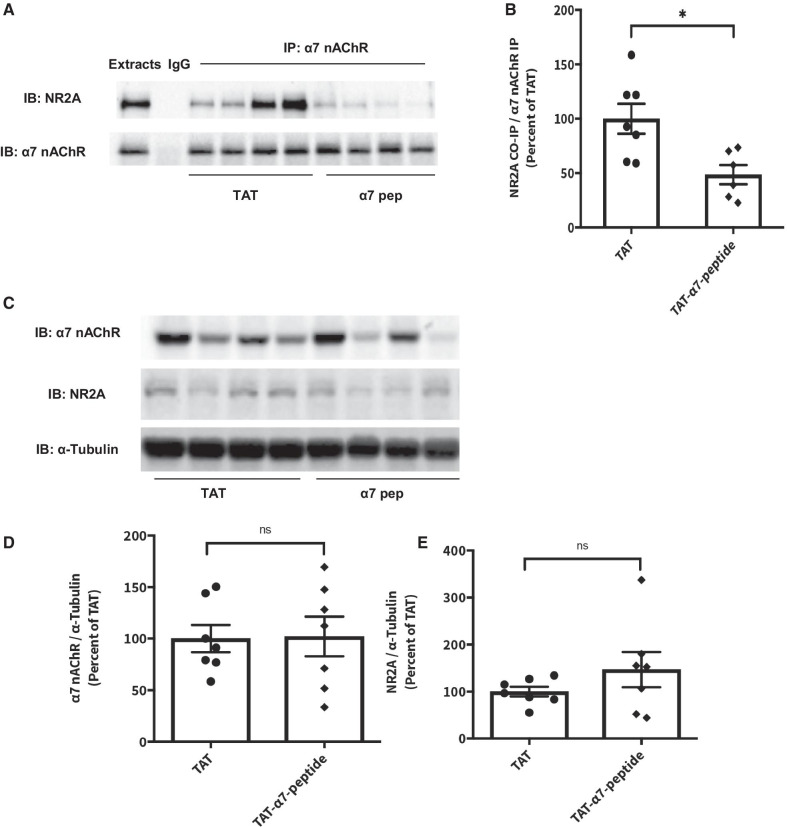
TAT-α7-peptide is able to decrease the α7nAChR–NR2A interaction in rat hippocampus. **A** Representative blot image of co-immunoprecipitation which shows that TAT-α7-peptide, but not TAT, decreased the α7nAChR–NR2A interaction in rat hippocampus. Tissue extract was used as positive control and IgG isotype was used to rule out the non-specific interaction between the antibody and protein of interest. **B** Densitometric analysis of the level of NR2A co-immunoprecipitated by α7nAChR antibody in hippocampal lysate of rats injected with TAT, or TAT-α7-peptide. The level of co-immunoprecipitated NR2A (NR2A Co-IP) was normalized after being divided by the level of precipitated α7nAChR (α7nAChR IP). Results for each sample are presented as the percentage of TAT group. *p < 0.05 as compared to TAT group, n = 7, Student’s t-test was performed to examine the statistical significance. Data were shown as mean ± SEM. **C** Representative Western blot image shows no difference in expression levels of α7nAChR and NR2A in lysate of rat hippocampus injected with TAT, or TAT-α7-peptide. α-Tubulin was used as a loading control. **D** Densitometric analysis of the expression levels of α7nAChR in hippocampal lysate of rats injected with TAT, or TAT-α7-peptide. The level of α7nAChR was normalized after being divided by the level of α-Tubulin. Results for each sample are presented as the percentage of the TAT samples. n = 7, Student’s t-test was performed to examine the statistical significance (*ns* no statistical significance). Data were shown as mean ± SEM. **E** Densitometric analysis of the expression levels of NR2A in hippocampal lysate of rats injected with TAT, or TAT-α7-peptide. The level of NR2A was normalized after being divided by the level of α-Tubulin. Results for each sample are presented as the percentage of the TAT samples. n = 7, Student’s t-test was performed to examine the statistical significance. Data were shown as mean ± SEM

We used co-immunoprecipitation assays to confirm that the α7nAChR–NR2A interaction is disrupted by the interfering peptide in rat brain tissues after the FST and locomotor activity tests. The hippocampus and striatum, brain regions implicated in the pathobiology of MDD, were collected after the behavioural tests were complete. As shown in Fig. 3A, B, the levels of the α7nAChR–NR2A protein complex are significantly lower in hippocampus of animals injected with TAT-α7-peptide as compared to those in the TAT-only control. However, the levels of α7nAChR–NR2A interaction in striatum were not significantly altered by TAT-α7-peptide (Additional file 1: Figure S1A, B). The expression levels of both α7nAChR and NR2A are not significantly different between TAT- and TAT-α7-peptide-injected groups in either hippocampus or striatum (Fig. 3C–E and Additional file 1: Figure S1C–E).

### Disruption of α7nAChR–NR2A interaction by TAT-α7-peptide increases ERK1 phosphorylation in rat hippocampus

We previously demonstrated that activation of α7nAChR facilitates NMDA receptor function via the α7nAChR–NR2A interaction, and that the TAT-α7-peptide can block this enhanced NMDA receptor-mediated current [41]. This phenomenon resembles the inhibition of over-activation of NMDA receptors which could potentially ameliorate depression-like behaviours. To investigate the mechanism by which disruption of the α7nAChR–NR2A complex results in anti-depressant effects, we examined downstream effector proteins. We used Western blots to determine if the TAT-α7-peptide alters phosphorylation of ERK1 or expression of BDNF in rat hippocampal tissues after the FST and locomotor activity tests. We chose to focus on ERK1 and BDNF based on their association with the α7nAChR–NR2A interaction [40] and MDD [45].

**Fig. 4:**
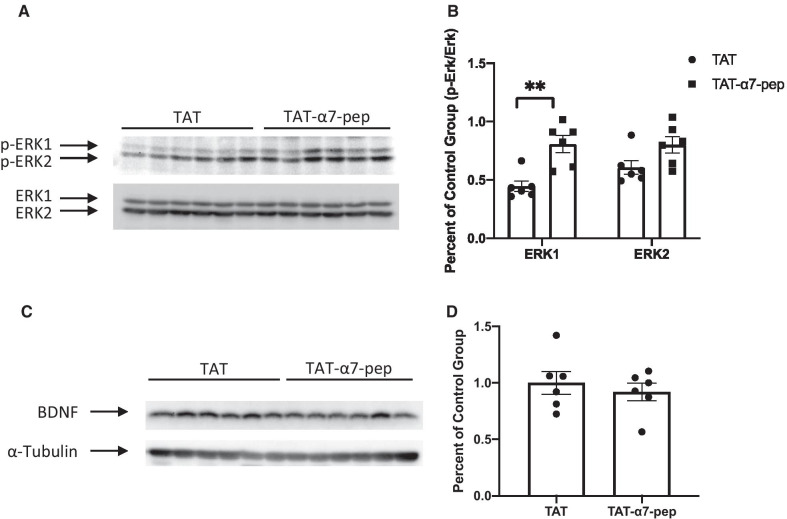
TAT-α7-peptide increases ERK1 phosphorylation, but not BDNF expression levels in rat hippocampal tissue. **A** Representative Western blots of the levels of phosphorylated ERK1/2 in rat hippocampal tissues with TAT peptide and TAT-α7-peptide. The levels of phosphorylated ERK1/2 were normalized after being divided by the level of ERK1/2. **B** Densitometric analysis of the levels of phosphorylated ERK1 in TAT-α7-peptide group is significantly higher than TAT group (Student’s t-test, **p < 0.01, n = 6). Data were shown as mean ± SEM. **C** Representative Western blots of the levels of BDNF in rat hippocampal tissues with TAT peptide and TAT-α7-peptide. The levels of BDNF were normalized after being divided by the levels of α-Tubulin. **D** Densitometric analysis of the levels of BDNF in TAT-α7-peptide group is not significantly different from those in TAT group (Student’s t-test, n = 6). Data were shown as mean ± SEM

In animals exposed to the FST, phosphorylated ERK1 was significantly higher in the hippocampus of animals that received TAT-α7-peptide (Fig. 4A, B). BDNF expression in hippocampus was not significantly altered by TAT-α7-peptide (Fig. 4C, D). To examine whether the FST could have altered phosphorylation and masked the effects of the interfering peptide, we measured ERK1 phosphorylation in animals that were not exposed to the FST. We found that the FST did not alter ERK1 phosphorylation FST (Additional file 1: Figure S2A, B). These data suggest that the antidepressant-like effects of TAT-α7-peptide may be mediated by increased ERK1 phosphorylation.

## Discussion

Our main finding is that disrupting the α7nAChR–NR2A interaction with TAT-α7-peptide has effects similar to existing antidepressants on the FST. This effect was apparent after only one dose, which did not cause significant hyperactivity. ERK1 phosphorylation was increased in conjunction with the FST, suggesting a potential mechanistic pathway. Our data identify the α7nAChR–NR2A protein complex as a potential novel treatment target for depression.

Another significant finding in this study is the observation that the α7nAChR–NR2A protein complex was detected in the striatum. Previously we had observed this protein complex only in the hippocampus. However, it is curious that the TAT-α7-peptide disrupted the α7nAChR–NR2A interaction in the hippocampus but not the striatum. The most likely explanation is anatomical; in rats, the hippocampus is directly exposed to the cerebrospinal fluid of the lateral ventricle [46, 47]. Thus, an *i.c.v.* injection would deliver peptide directly to the ventricular surface of the hippocampus. Conversely, the only striatal structure with ventricular contact is the part of the dorsal striatum, with the remaining large structures of the striatum having no contact with the CSF [46, 48]. Therefore, we hypothesize that *i.c.v.* injections of TAT-α7-peptide do not reach the majority of striatal tissue.

It was unexpected that TAT α7-peptide increases ERK1 phosphorylation in the hippocampus. This seems to contradict previous data that TAT-α7-peptide significantly reduced phospho-ERK1 and phospho-ERK2 levels induced by a nicotine-associated cue [40]. One possible explanation is that our previous experiments used rats that had reinstated nicotine seeking, while the current data are from rats exposed to the FST and never to nicotine. We do not have data showing that reinstated nicotine seeking enhances ERK1/2 phosphorylation. But we did observe that chronic nicotine treatment increased the amount of α7nAChR–NR2A protein complex ([40], Fig. 1F, G). Thus, TAT-α7-peptide may not directly reduce the phosphorylation of ERK1/2, but rather normalized ERK1/2 phosphorylation that had been altered by reinstatement of nicotine seeking.

In our study, we used the FST to screen for antidepressant-like effects of the TAT-α7-peptide. We are aware that the FST remains controversial as an antidepressant screening test, and the theoretical justification remains problematic since there is no human equivalent of this animal paradigm [49]. An important direction for further experiments would include other behavioural tests relevant to depression and antidepressant medication effects such as sucrose preference and Crawley’s three-chamber social interaction test. It would also be useful to study the effects of our peptide on animals with depressive-like states induced by paradigms such as chronic mild stress or social defeat.

To confirm that the antidepressant effects of TAT-α7-peptide were mediated by disruption of the α7nAChR–NR2A protein complex, but not by altered levels of the constituent proteins, we used Western blots to measure α7nAChR and NR2A protein levels. We found a large variance in the amounts of these proteins, especially α7nAChR (Fig. 3). We do not have data to explain this observation, but speculate that this variance could have arisen from several possible sources. It is possible that animals had differential sensitivity to the FST. It is common to observe variance in response to stressful environmental manipulations, both at the behavioural and molecular level, and this is the most likely explanation here [50]. In addition, our experiments were performed with the outbred rat Sprague–Dawley strain [51], not genetically inbred mice as in some of our previous experiments involving behaviour and protein complexes [42]. The genetic variation inherent in an outbred rat strain could have contributed to a heterogeneous response to the FST. It is also possible that variation in experimental conditions such as the surgical procedures to implant the cannula could have led to variation in protein expression [52, 53].

We examined the level of BDNF and whether the TAT-α7-peptide might alter BDNF levels in conjunction with antidepressant-like effects on the FST. BDNF has been extensively studied in the context of depression and animal models of stress [54], as well as in other brain disorders such as schizophrenia [55]. In particular, a prominent hypothetical model for depression involves the epigenetic mechanism of histone methylation that transduces environmental stress into decreased BDNF expression. Antidepressant medications like the imipramine used in our study, can restore BDNF levels through histone acetylation in the BDNF locus [56]. Thus, we tested the hypothesis that BDNF might also be connected to the α7nAChR–NR2A protein complex, but did not observe any change in BDNF levels.

In summary, we report that disrupting the α7nAChR–NR2A protein complex with TAT-α7-peptide has antidepressant-like effects on the FST. This identifies a novel potential target for treating depression. Our data also suggest that this protein complex could play a role in the pathophysiology of depression. Of course, many additional experiments are required to understand the molecular mechanisms that underlie our initial observations. Nevertheless, given the magnitude of the global disease burden caused by depression, and the large number of patients with treatment-resistant depression, it is important to pursue new pathways and targets for treatment.

## Supplementary Information

**Additional file 1: Figure S1.** TAT-α7-peptide does not change the α7nAChR–NR2A interaction in rat striatum. **A** Representative blot image of co-immunoprecipitation which shows that the levels of α7nAChR–NR2A interaction in rat striatum are not different between rats injected with TAT and TAT-α7-peptide. Tissue extract was used as positive control and IgG isotype was used to rule out the non-specific interaction between the antibody and protein of interest. **B** Densitometric analysis of the level of NR2A co-immunoprecipitated by α7nAChR antibody in striatal lysate of rats injected with TAT, or TAT-α7-peptide. The level of co-immunoprecipitated NR2A (NR2A Co-IP) was normalized after being divided by the level of precipitated α7nAChR (α7nAChR IP). Results for each sample are presented as the percentage of TAT group. n = 4, Student’s t-test was performed to examine the statistical significance (ns—no statistical significance). Data were shown as mean ± SEM. **C** Representative Western blot image shows no difference in expression levels of α7nAChR and NR2A in striatal lysate of rats injected with TAT, or TAT-α7-peptide. α-Tubulin was used as a loading control. **D** Densitometric analysis of the expression levels of α7nAChR in striatal lysate of rats injected with TAT, or TAT-α7- peptide. The level of α7nAChR was normalized after being divided by the level of α-Tubulin. Results for each sample are presented as the percentage of the TAT samples. n = 4, Student’s t-test was performed to examine the statistical significance. Data were shown as mean ± SEM. **E **Densitometric analysis of the expression levels of NR2A in striatal lysate of rats injected with TAT, or TAT-α7-peptide. The level of NR2A was normalized after being divided by the level of α-Tubulin. Results for each sample are presented as the percentage of the TAT samples. n = 4, Student’s t-test was performed to examine the statistical significance. Data was shown as mean ± SEM. **Figure S2.** FST does not change the phosphorylation level of ERK1/2. **A** Representative blot image showing the expression of phosphorylated ERK1/2 and phosphorylated ERK1/2 in the rat hippocampus. **B** Densitometric analysis of the percent phosphorylated ERK1/2 in rat hippocampus (n = 4 for both non-swim and swim group, data were normalized after being divided by the level of total ERK1/2). Student’s t-test was performed to examine the statistical significance, and not statistical significance was observed (p = 0.4920 for percent of phosphorylated ERK1 and p = 0.9912 for percent of phosphorylated ERK2). Data were shown as mean ± SEM.

## Data Availability

The datasets used and/or analyzed during the current study are available from the corresponding author on reasonable request.

## Abbreviations

MDD: Major depressive disorder
nAChR: Nicotinic acetylcholine receptor
NMDARs: NMDA glutamate receptors
FST: Forced swim test
ERK: Extracellular signal-regulated kinase
SSRIs: Selective serotonin reuptake inhibitors
TCA: Tricyclic antidepressants
MAOIs: Monoamine oxidase inhibitors
CBT: Cognitive-behavioral therapy
IPT: Interpersonal therapy
AChE: Acetylcholine esterase
TST: Tail suspension test
PFC: Prefrontal cortex
CSF: Cerebrospinal fluid
BCA: Bicinchoninic acid
SEM: Standard error of the mean
BDNF: Brain-derived neurotrophic factor
