# Supplementary figures and images for: Disrupting the α7nAChR–NR2A protein complex exerts antidepressant-like effects
Source: Mol Brain. 2021 Jul 5;14:107. doi: 10.1186/s13041-021-00817-3 (PMC8256601; doi:10.1186/s13041-021-00817-3)

## Slide 1
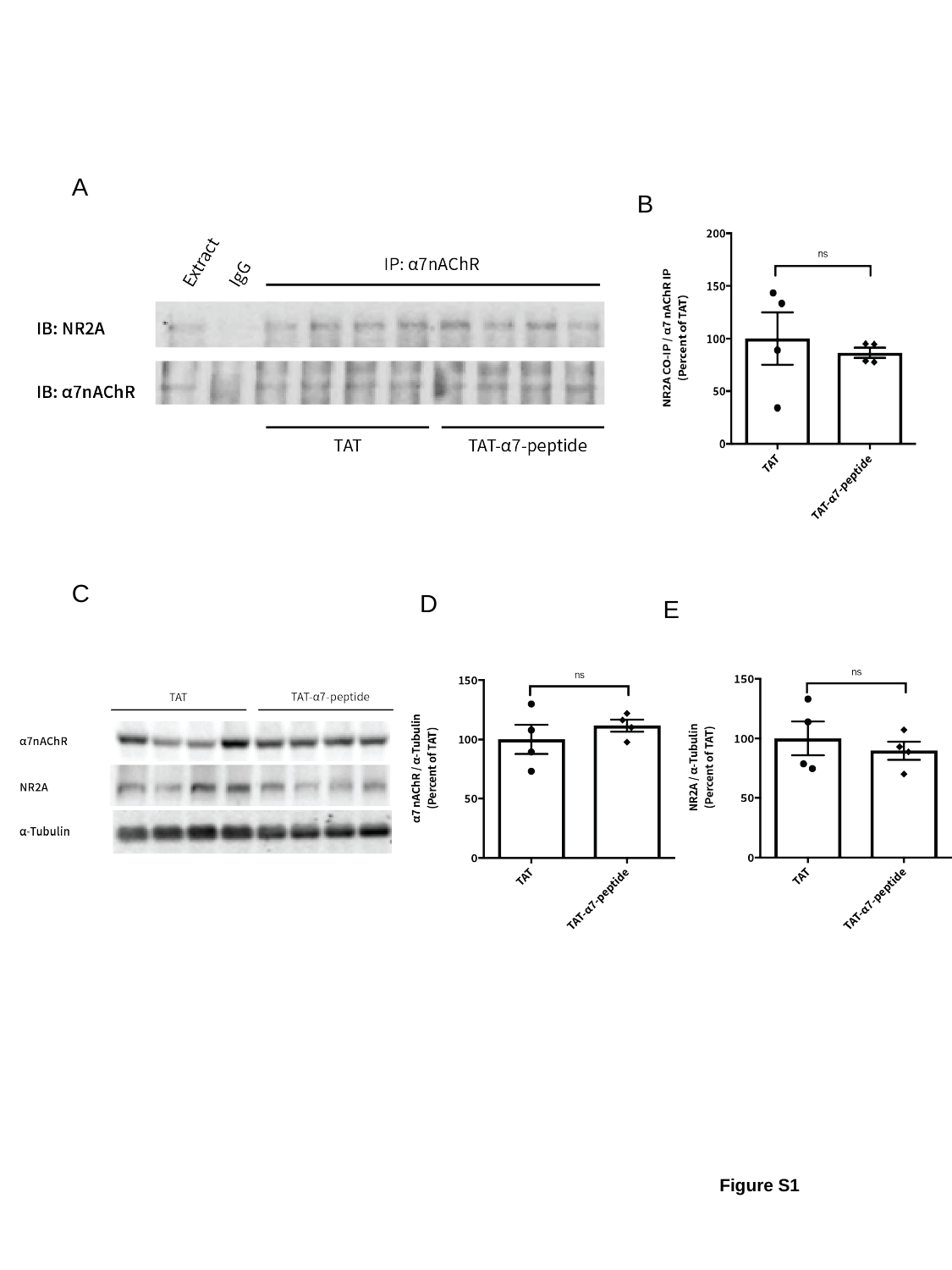

A
B
C
D
E
Figure S1

## Slide 2
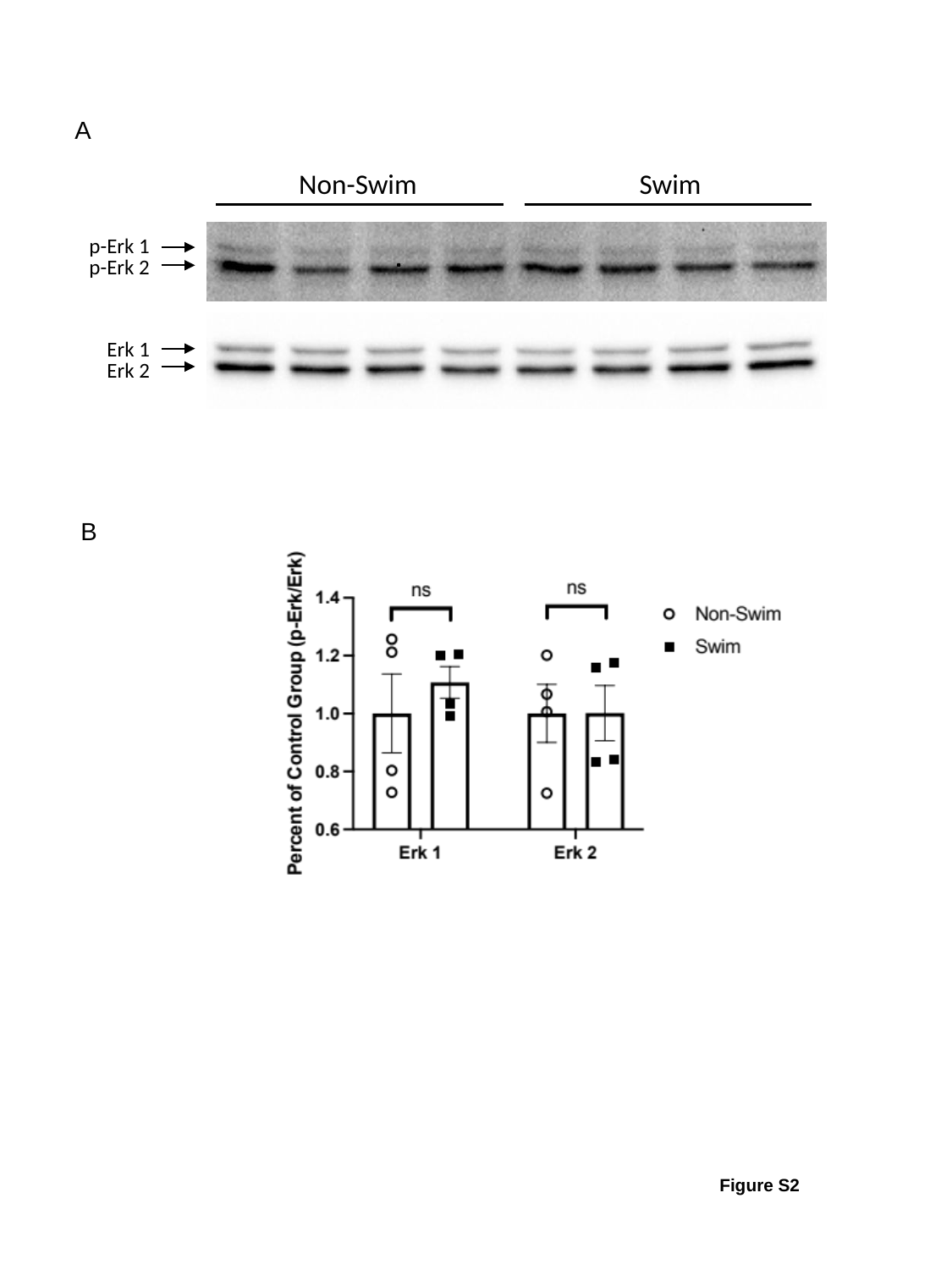

A
Non-Swim
Swim
p-Erk 1
p-Erk 2
Erk 1
Erk 2
B
Figure S2

Supplement: Supplementary file 1 — Additional file 1: Figure S1. TAT-α7-peptide does not change the α7nAChR–NR2A interaction in rat striatum. A Representative blot image of co-immunoprecipitation which shows that the levels of α7nAChR–NR2A interaction in rat striatum are not different between rats injected with TAT and TAT-α7-peptide. Tissue extract was used as positive control and IgG isotype was used to rule out the non-specific interaction between the antibody and protein of interest. B Densitometric analysis of the level of NR2A co-immunoprecipitated by α7nAChR antibody in striatal lysate of rats injected with TAT, or TAT-α7-peptide. The level of co-immunoprecipitated NR2A (NR2A Co-IP) was normalized after being divided by the level of precipitated α7nAChR (α7nAChR IP). Results for each sample are presented as the percentage of TAT group. n = 4, Student’s t-test was performed to examine the statistical significance (ns—no statistical significance). Data were shown as mean ± SEM. C Representative Western blot image shows no difference in expression levels of α7nAChR and NR2A in striatal lysate of rats injected with TAT, or TAT-α7-peptide. α-Tubulin was used as a loading control. D Densitometric analysis of the expression levels of α7nAChR in striatal lysate of rats injected with TAT, or TAT-α7- peptide. The level of α7nAChR was normalized after being divided by the level of α-Tubulin. Results for each sample are presented as the percentage of the TAT samples. n = 4, Student’s t-test was performed to examine the statistical significance. Data were shown as mean ± SEM. E Densitometric analysis of the expression levels of NR2A in striatal lysate of rats injected with TAT, or TAT-α7-peptide. The level of NR2A was normalized after being divided by the level of α-Tubulin. Results for each sample are presented as the percentage of the TAT samples. n = 4, Student’s t-test was performed to examine the statistical significance. Data was shown as mean ± SEM. Figure S2. FST does not change the phosphorylation [file 13041_2021_817_MOESM1_ESM.pptx]
